# ReVac: a reverse vaccinology computational pipeline for prioritization of prokaryotic protein vaccine candidates

**DOI:** 10.1186/s12864-019-6195-y

**Published:** 2019-12-16

**Authors:** Adonis D’Mello, Christian P. Ahearn, Timothy F. Murphy, Hervé Tettelin

**Affiliations:** 1 Department of Microbiology and Immunology, Institute for Genome Sciences, University of Maryland School of Medicine, Baltimore, MD 21201 USA; 2 Department of Microbiology and Immunology, University at Buffalo, the State University of New York, Buffalo, NY USA; 3 Clinical and Translational Research Center, University at Buffalo, the State University of New York, Buffalo, NY USA; 4 Division of Infectious Disease, Department of Medicine, University at Buffalo, the State University of New York, Buffalo, NY 14203 USA

**Keywords:** Reverse vaccinology, Vaccines, Antigen scoring, Orthology, Core genome, Bacterial, Pan-genome

## Abstract

**Background:**

Reverse vaccinology accelerates the discovery of potential vaccine candidates (PVCs) prior to experimental validation. Current programs typically use one bacterial proteome to identify PVCs through a filtering architecture using feature prediction programs or a machine learning approach. Filtering approaches may eliminate potential antigens based on limitations in the accuracy of prediction tools used. Machine learning approaches are heavily dependent on the selection of training datasets with experimentally validated antigens (positive control) and non-protective-antigens (negative control). The use of one or few bacterial proteomes does not assess PVC conservation among strains, an important feature of vaccine antigens.

**Results:**

We present ReVac, which implements both a panoply of feature prediction programs without filtering out proteins, and scoring of candidates based on predictions made on curated positive and negative control PVCs datasets. ReVac surveys several genomes assessing protein conservation, as well as DNA and protein repeats, which may result in variable expression of PVCs. ReVac’s orthologous clustering of conserved genes, identifies core and dispensable genome components. This is useful for determining the degree of conservation of PVCs among the population of isolates for a given pathogen. Potential vaccine candidates are then prioritized based on conservation and overall feature-based scoring. We present the application of ReVac, applied to 69 *Moraxella catarrhalis* and 270 non-typeable *Haemophilus influenzae* genomes, prioritizing 64 and 29 proteins as PVCs, respectively.

**Conclusion:**

ReVac’s use of a scoring scheme ranks PVCs for subsequent experimental testing. It employs a redundancy-based approach in its predictions of features using several prediction tools. The protein’s features are collated, and each protein is ranked based on the scoring scheme. Multi-genome analyses performed in ReVac allow for a comprehensive overview of PVCs from a pan-genome perspective, as an essential pre-requisite for any bacterial subunit vaccine design. ReVac prioritized PVCs of two human respiratory pathogens, identifying both novel and previously validated PVCs.

## Background

Reverse vaccinology pipelines use genome datasets to identify potential vaccine candidates (PVCs) based on in silico prediction of hallmark features of an ideal vaccine candidate antigen. These features include presence of epitopes exposed on the bacterial surface for host immune recognition, antigenicity, sequence conservation across isolates, and expression during infection [1, 2]. Since the development and application of reverse vaccinology to the case of Serogroup B meningococcus [3], its potential for growth has increased significantly with the advent of next-generation sequencing techniques, development of bioinformatic tools for multi-genome analyses, protein functional predictions, and high throughput protein expression platforms [4]. These advances in technology offer an opportunity to generate new reverse vaccinology programs that accurately predict candidate bacterial proteins for use in subunit-based vaccines.

Several tools have been developed for antigen prediction and vaccine candidate identification, including NERVE, Jenner-Predict, Vaxign, VaxiJen, VacSol, and Bowman-Heinson [5]. These tools typically follow either filtering or machine learning algorithms. The filtering workflows utilize a single program for each feature prediction and filter out proteins at each stage. A limitation of the filtering architecture is the potential of elimination of vaccine candidates from further analyses, in the event of a false negative prediction by any given bioinformatic tool. The machine learning workflows use datasets of known PVCs and negative controls to classify antigens and non-antigens through a probability score. To date, tools applying either of the two approaches consider protein sequences exclusively. An extensive review of all these workflows can be found in Dalsass et al. [5].

Here we describe ReVac, a computational pipeline for prediction and prioritization of protein-based bacterial vaccine candidates for experimental verification. ReVac surveys several genomes, using multiple independent tools for predictions of the same feature, to assess a large panel of protein features and sequence conservation. ReVac also scans both the protein and DNA sequences of genes for repeat sequences that could mediate phase variation (gene on/off switching) or protein structure variations, attributes that are typically not desirable in a candidate for vaccine development [6]. ReVac compiles all data across various features, at the protein and nucleotide level, from several bacterial genomes, into one tab-delimited output file. It also scores each protein based on each individual feature in parallel, without eliminating any candidate from analyses. A general problem in reverse vaccinology is that most workflows predict hundreds of proteins as vaccine candidates, rendering experimental verification assays cumbersome [5]. Although some provide a ranking of candidates based on sequence similarity with curated epitopes [7], this approach does not promote the discovery of new types of candidates from different bacteria. ReVac uses its own scoring scheme for the output of each feature prediction tool that is part of its workflow. The scoring scheme was developed, based on manually observing trends of feature predictions, of control datasets of known antigens and non-antigens. These control datasets were obtained from various antigen/epitope databases of predicted and experimentally curated proteins, namely Protegen, AntigenDB, Vaxign’s control datasets, ePSORTB. We supplemented these publicly available datasets with known antigens from our *Moraxella catarrhalis* and non-typeable *Haemophilus influenzae* (NTHi) datasets [8–13]. These control datasets consist of DNA and protein sequences from various Gram-positive and Gram-negative species, which were run through ReVac (Additional file 1), and the corresponding scoring scheme is shown in Table 2.

The final output of ReVac consists of a list of predicted vaccine candidates sorted based on their ReVac scores, an aggregate scoring scheme that combines individual feature weights assigned to each of the candidates’ features. This allows the user to consider candidates by perusing those with the highest ReVac scores. Importantly, ReVac accounts for strain to strain variation when prioritizing top candidates by generating clusters of orthologous genes across all genomes of the species of interest. ReVac displays average scores of gene conservation for each ortholog cluster to provide an estimate of variation. These two innovations in reverse vaccinology application allow for selection of a manageable number of conserved PVCs for experimental verification and vaccine development.

## Results

### ReVac workflow

**Fig. 1:**
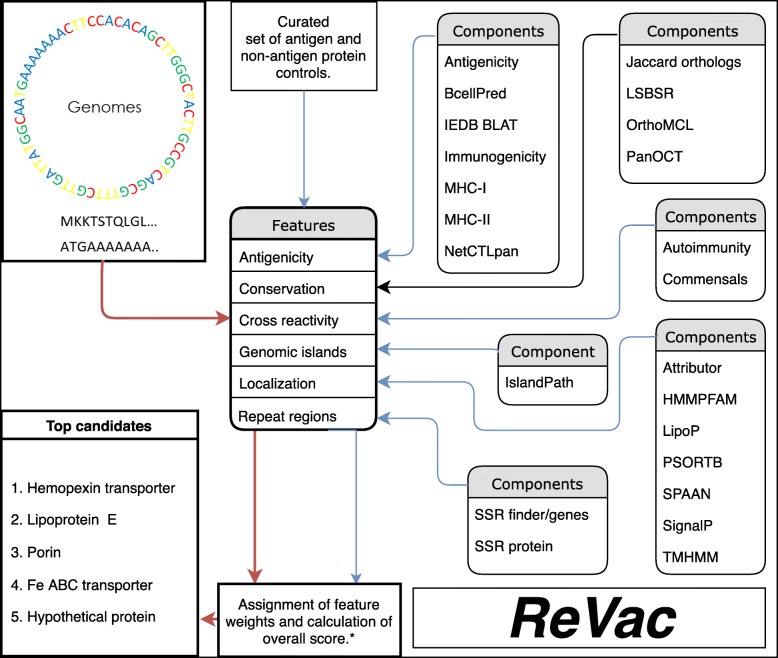
Schematic of the ReVac workflow, its components and underlying features. Blue arrows indicate the components where control datasets were used to develop the scoring algorithm. Red arrows indicate a user’s input query dataset, which runs through all components and the scoring algorithm, to output a list of prioritized candidates for the supplied species. Scoring based on core genes or orthology components is indicated by the black arrow

The ReVac pipeline uses the Ergatis workflow management system to analyze all data on distributed computer clusters [14]. Figure 1 shows the overall workflow and components of ReVac. Parallel computing allows ReVac to run efficiently while performing predictions on entire collections of input genomes. Analysis is launched using a list of GenBank-formatted genomes as input. ReVac’s foundation components convert the GenBank files to formats suiting each predictive tool’s input, as necessary. Amino acid and nucleotide gene sequence FASTA files, as well as annotation General Feature Format (GFF), files are created. Their content is then binned into smaller subsets of data that are submitted as parallel batches on the compute cluster.

ReVac utilizes several bioinformatic tools for its protein or nucleotide feature predictions (Fig. 1, Table 2, and Methods) that are grouped into the following categories: subcellular localization, antigenicity & immunogenicity, conservation & function, exclusion features, genomic islands, and foundation components. Subcellular localization contains tools predicting overall protein localization from the analyses of lipoprotein signal, transmembrane helices, signal peptide presence, adhesin potential, and HMM (Hidden Markov Model) domains associated with surface exposure. Antigenicity & immunogenicity covers Major Histocompatibility Complex (MHC) class I and II binding capabilities, B-cell epitope presence, overall MHC immunogenicity and a BLAT (BLAST-Like Alignment Tool) [15] alignment with known experimentally verified epitopes, acquired from the Immune Epitope Database & Analysis Resource (IEDB) [16]. Conservation & function applies 4 different methods for generating clusters of orthologs, and implements a tool that updates annotations and assigns Gene Ontology (GO) terms [17]. Exclusion features determine protein similarity to *Homo sapiens* proteins (risk of autoimmunity) and a user-defined list of commensal organisms (to address the risk of depleting the microbiome), as well as the prediction of amino acid and/or nucleotide repeats that mediate phase variation. Genomic Islands (GI) prediction informs whether or not a gene is carried within a putative mobile element and therefore transmissible between isolates or species. Lastly, foundation components refer to all tools involved in file format conversion, input data generation and text processing. The implementation of multiple prediction tools and scoring schemes for most of the features considered compensates for each individual tools’ potential for false negative/positive predictions. Given these attributes, ReVac offers an innovative and comprehensive workflow design for reverse vaccinology.

**Fig. 2:**
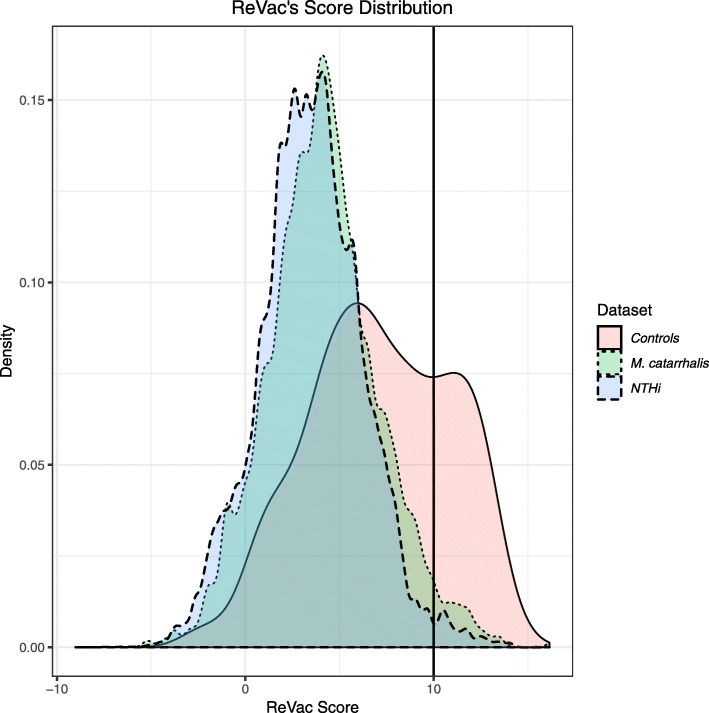
A density plot showing the scores for all sequences run through ReVac, and the cutoff for our *M. catarrhalis* and NTHi datasets

Outputs from ReVac’s components are systematically converted into tab-delimited format and grouped by protein IDs or locus tags derived from the GenBank files. This is achieved using in-house Perl scripts, to generate ReVac’s initial gene feature summary table. This table is then parsed using ReVac’s scoring algorithm (Table 2) and a final score-sorted summary table is reported. These two tables include results for all genes provided as input without eliminating any potential candidates. To look for highly conserved core vaccine candidates, the scored summary table is further parsed for overall protein conservation, comparing all 4 orthology methods used, across all genomes. ReVac then refines the list of PVCs for those with ReVac scores comprised of a distribution of ideal PVCs feature (i.e where the ReVac scores were penalized by a total of less than 10% of its overall score, due to the presence of undesirable PVC’s scoring features). All clusters are then grouped and given an ortholog ID. Their annotation, average, minimum and maximum ReVac scores are reported at an ortholog cluster level. Based on scores observed for positive and negative controls we used, clusters harboring average scores higher than a ReVac score of 10 with minimum variation (based on the reported average, minimum and maximum) in the scores across the cluster, are ranked as top PVCs. A higher score cutoff can be chosen by the user to further reduce the number of prioritized candidates. Here, 10 was chosen as the cutoff for our NTHi and *M. catarrhalis* datasets, as it was observed that the frequency of non-antigens was higher below this value (Fig. 2, left peak of Controls), while the frequency of antigens formed a second distinct peak for scores 10 and higher (Fig. 2, right peak of Controls) (See also Additional file 1). Implementation of higher cutoffs to focus the list of candidates in a separate small table does not eliminate any candidates from the complete scored table. Other candidates can be selected by scanning the full table that shows PVCs in ranked order and evaluating the relative importance of features that may have diminished their overall score.

### Control datasets used for development of the scoring scheme

The control datasets used in ReVac comprise a total of 564 proteins acquired from Vaxign, Protegen and AntigenDB [8, 9, 12], as well as our manually curated list of NTHi and *M. catarrhalis* antigens [10, 11]. Where possible, protein identifiers (IDs) from these three public databases were systematically converted to Uniprot unique IDs for consistency and ease of access to protein characteristics (Additional file 1: Sheet 3). Because ReVac is the first pipeline to consider nucleotide features associated with candidate antigens, we also obtained closely related nucleotide sequences for all public candidates by retrieval of best TBLASTN [18] hits against the National Center for Biotechnology Information (NCBI) nt database of non-redundant nucleotide sequences (all hits were to the respective species). Among other features, nucleotide sequences provided information on simple sequence repeats (SSRs) that may mediate phase variation.

Since these databases contained some of the same sequences or different alleles of the same antigens, we used OrthoMCL [19] to identify their orthologs (Additional file 1). Of the 564 proteins, 376 were assigned to 102 clusters by OrthoMCL. As we were interested in the scores across all alleles of an antigen, we included all 564 in our analysis. The 564 proteins were split into 136 Gram-positive and 428 Gram-negative datasets using the species and associated Gram stain information provided from their respective databases. We also used the species hits from the TBLASTN results for this purpose. These two datasets were then run on two pipelines, each with relevant Gram-positive or Gram-negative parameters required for some of the tools incorporated in ReVac. Of the 564, 41 were unique non-antigens from Vaxign [9] and were included to assess their scores relative to our weighing scheme. All proteins from control datasets were run through the workflow (except orthology given the wide range of species represented) for development of the scoring scheme (Table 2.). Inspection of positive and negative control proteins enabled optimization and implementation of score boosting for desired features carried by real antigens, as well as maximum thresholds of penalization in the case of autoimmunity and SSRs, as described in the Methods. Summary tables from ReVac runs on all datasets are available in Additional files 1, 2 and 3.

A subset of the controls used is presented in Table 1 to illustrate the process of optimizing feature scoring. The scores for each component were developed by observing trends in the predicted features of all the tools and their correlation to whether the control protein was antigenic or non-antigenic. For example, the first 2 antigens from Table 1, the pertactin autotransporter from *Bordetella pertussis* and the peptidoglycan-associated outer membrane lipoprotein (P6) from NTHi, have overall subcellular localization predictions suggesting surface exposure, consistent with previous experimental findings [11, 20, 21]. The tools that accurately predicted these features were assigned positive weights (shown in Table 2) to identify other proteins displaying these features. In events when multiple tools show strong predictions of surface localization, the ReVac score is boosted as it was observed in multiple antigens from the dataset, and these features indicate a strong potential vaccine candidate. As for the tools that provided no features for these two antigens, they were not weighted negatively as they weren’t necessary for surface exposure in the case of these two antigens but may be relevant to other proteins. We see this in the case of the *Streptococcus agalactiae* antigen, C protein alpha-antigen [22], where the presence of transmembrane helices and adhesin features were predicted in the protein. These tools were also assigned positive weights for identification of these features in other proteins, based on their observed frequency within the control dataset (Table 2). Since some of the tools have no conclusive feature predictions for certain sequences, such antigens have lower overall ReVac scores.

**Table 1:** Examples of control proteins used to develop the scoring scheme, and a summary of the outputs from each of ReVac’s components

General Information							
No	ReVac Score	Score Breakdown	Organism	Gram Stain	Type		
1	14.853	15.253–0.400	*Bordetella pertussis*	–	Antigen		
2	13.709	13.709–0.000	Non-typable *Hemophilus influenzae*	–	Antigen		
3	9.049	9.049–0.000	*Moraxella catarrhallis*	–	Antigen		
4	8.192	8.192–0.000	*Streptococcus agalactiae* A909	+	Antigen		
5	6.791	6.791–0.000	*Streptococcus pneumoniae*	+	Antigen		
6	6.32	6.520–0.200	*Neisseria meningitidis* LNP21362	–	Antigen		
7	5.768	7.768–2.000	*Streptococcus pneumoniae*	+	Non Antigen		
8	2.475	5.542–3.066	*Clostridium perfringens* str. 13	+	Non Antigen		
Surface Exposure Predictions							
No.	PSORTB Localization	LipoProtein	Transmembrane Helices	Signal Peptide	SPAAN adhesin ratio	HMM mapping to surface exposed database	Annotation/GO Terms
1	OuterMembrane	SignalPeptidase I	None	MNMSLSRIVKAAPLRRTTLAMALGALGAAPAAHA	None	Positive	outer membrane autotransporter barrel|GO:0009405,GO:0015474,GO:0045203,GO:0046819
2	OuterMembrane	SignalPeptidase II	None	MNKFVKSLLVAGSVAALAACSSSNNDA	None	Positive	peptidoglycan-associated lipoprotein|GO:0009279
3	None	SignalPeptidase II	None	MQFSKSIPLFFLFSIPFLA	None	Positive	Bacterial extracellular solute-binding protein
4	Cellwall	SignalPeptidase I	1	None	0.782535	Positive	hypothetical protein
5	Extracellular	Intracellular	None	None	None	None	Thiol-activated cytolysin family protein|GO:0015485,GO:0009405
6	Periplasmic	SignalPeptidase II	None	MFKRSVIAMACIFALSACG	None	None	Transferrin binding family protein|GO:0016020
7	None	Intracellular	None	None	None	None	Capsular polysaccharide synthesis family protein
8	None	Intracellular	None	None	None	None	shikimate dehydrogenase ec::1.1.1.25|GO:0004764,GO:0009423
Antigenicity Predictions^a^							
No.	Antigenicity	B cell epitopes	MHC I binding	MHC II binding	MHC binding + Antigen Processing	Immunogenicity within MHC complex	Alignment to curated epitopes
1	45.05%	15.16%	94.07%	100.00%	61.10%	13.08%	26.92%
2	30.72%	5.23%	96.73%	94.12%	79.08%	17.65%	99.35%
3	44.02%	16.03%	94.57%	100.00%	69.57%	23.91%	None
4	50.40%	38.97%	83.10%	90.46%	43.34%	1.79%	None
5	43.74%	13.80%	95.33%	98.94%	73.04%	12.10%	22.08%
6	30.33%	15.16%	81.56%	86.68%	46.93%	1.84%	None
7	48.94%	4.61%	96.81%	100%	81.91%	30.85%	None
8	34.32%	7.75%	96.31%	98.89%	77.49%	16.61%	None
Adverse Features							
No.	Autoimmunity with humans	Repeat regions genes & copy number	Repeat regions proteins & copy number				
1	None	None	|APAGGAVPGG 2||PQP 3|				
2	None	None	None				
3	None	None	None				
4	None	None	None				
5	None	None	None				
6	None	None	|ARFRRS 2|				
7	None	None	None				
8	3.32%	None	None				

^a^Percents are relative to the length of the amino acid sequence

**Table 2:** List of all the programs run in ReVac and their predicted features, with the scoring scheme for each programs output. Additional scoring descriptions based on outputs from multiple programs are listed at the bottom

Module (Reference)	Gene property	Evidence	Output	Scoring weight (points)	Example Protein (*M. catarrhalis* NAO366_1291)	Example Feature	Example Weight	Example Cumulative Score
PSORTb* [13]	Surface exposure^	Sub-cellular localization	Surface localization prediction	+ 1 if surface exposed	9.52|OuterMembrane	Positive surface exposure	1	1
				−1 if cytoplasmic				
LipoP [14]	Surface exposure^	Lipoprotein motif	Presence or absence of a motif	1 or 0	SpI|18.809	Positive for lipoprotein motif	1	2
TMHMM [15]	Surface exposure^	Transmembrane spans	Number of helices	If surface exposed < 2: + 0.5	1	Presence of 1 TMH	0.5	2.5
				02:00.0				
				3: −0.2				
				≥4: −2				
				If cytoplasmic				
				−2				
SignalP [16]	Surface exposure^	Signal peptide	Signal peptide	+ 1 for presence	MNKTSTQLGLLAVSVSLIMASLPAHA	Signal peptide present	1	3.5
SPAAN [17]	Surface exposure^	Adhesin protein	Adhesin protein score	+ 0.5 if above cutoff score (default 0.75)	0.907057	Predicted Adhesin	0.5	4
Surface HMMs [18]^a^	Surface exposure Function	HMM for motif or function	HMM title and score	0.5	None	No HMM alignment	0	4
Antigenic [19]	Antigenicity	Antigenic epitopes	Peptides, scores, protein coverage	0.5	QLGLLAVSVSLIMASLPAHAVYLDR|1.193|10(169)|41.73	Predicted antigenic region.	0.5	4.9173
				+ 0–1 proportional to coverage		41.73% of the protein is antigenic	0.4173	
Bcell Pred [20]	Antigenicity	B cell epitopes, 6 prediction methods combined	Number of peptides, protein coverage	+ 0–1 proportional to coverage	6(59)|14|14.57	Predicited B-cell Epitopes	0.1457	5.09809
				+ 0–1 proportional to total number of peptides of a given length per protein		14.57% predicted in 14 peptides of 7AA	14/(405–7 + 1) = 0.03509	
MHC class I [20]	Antigenicity	MHC-I epitopes	Number of peptides, protein coverage	+ 0–1 proportional to coverage if 80–90%	6(378)|124|73|93.33	Predicted MHC binding		6.41039
				+ 0–1 proportional to total number of peptides of a given length per protein		93.33% predicted in 124 peptides of 9AA	124/(405–9 + 1) = 0.3123	
				+ 1 if coverage is > = 90%			1	
NetCTLpan [20]	Antigenicity	MHC-I epitopes	Number of peptides, protein coverage	+ 0–1 proportional to coverage if 80–90%	12(334)|70|12|82.47	Predicted MHC binding	0.8247	7.41139
				+ 0–1 proportional to total number of peptides of a given length per protein		82.47% predicted in 70 peptides	70/(405–9 + 1) = 0.1763	
				+ 1 if coverage is > = 90%				
Immunogenicity (MHC-I) [20]	Antigenicity	MHC-I epitopes immunogenicity	Number of peptides, protein coverage	+ 0–1 proportional to coverage	7(76)|14|36|18.77	Predicted immunogenic region	0.1877	8.63435
				+ 0–1 proportional to total number of peptides of a given length per protein		14 peptides of 9AA	14/(405–9 + 1) = 0.035264	
				+ 1 if coverage is > = 10%			1	
MHC class II [20]	Antigenicity	MHC-II epitopes	Number of peptides, protein coverage	+ 0–1 proportional to coverage if 80–90%	2(404)|315|61|99.75	Predicted MHC-II binding		10.43995
				+ 0–1 proportional to total number of peptides of a given length per protein		99.75% predicted in 315 peptides of 15AA	315/(405–15 + 1) = 0.8056	
				+ 1 if coverage is > = 90%			1	
BLAT (IEDB^b^ database*) [20]	Antigenicity	Similarity to curated epitopes from IEDB	Protein coverage	+ 0–1 proportional to coverage	None	No hits to epitope database	0	10.43995
				+ 1 if coverage is > 70%				
Autoimmunity [5]	Autoimmunity	Similarity to human proteins	Protein coverage	+ 1 if no autoimmunity	None	No hits to Human	1	11.43995
				−2 *(0 to1) proportional to coverage				
				−2 if coverage is > 20%				
Autoimmunity Commensals [5]	Autoimmunity	Similarity to user-defined commensal organisms’ proteins	Protein coverage	+ 1 if no autoimmunity	3(39)|9.63	9.63% similarity to commensal	(0.0963)x(− 2) = −0.1926	11.24735
				−2 *(0 to1) proportional to coverage			(Negative feature)	
				−2 if coverage is > 20%				
SSR^d^ Finder [4]	Variability of expression	Phase variation	Number of simple sequence repeats	+ 1 if no SSR	None	No DNA SSR found	1	12.24735
				−0.5 for each SSR				
				−0.25 for each SSR in the promoter				
				−0.5 for each SSR with frameshift potential				
				−0.01 times the length of the SSR.				
SSR^d^ Finder Protein [4]	Variability of expression	Potential conformational shifts	Number of protein tandem repeats	−0.2 for each protein repeat, max penalty of 1.	None	No protein SSR found	0	12.24735
IslandPath [21]	Potential for horizontal gene transfer	Genomic Islands	Presence in a GI	−1 for each protein in a GI	None	Not present in a GI	0.5	12.74735
				+ 0.5 for absence				
Jaccard Clusters [22]†	Conservation	Orthologous clusters	Presence in an orthologous cluster	+ 1 for each protein in a COG in > = 90% of genomes in atleast one method	j_ortholog_cluster_3254|63	Present in > 90% of the genomes		
				−0.25 for each protein in a COG in < 90% of genomes				
PanOCT [23]†	Conservation	Orthologous clusters	Presence in an orthologous cluster	+ 1 for each protein in a COG in > = 90% of genomes in atleast one method	PanOCT_cluster_108|63	Present in > 90% of the genomes		
				−0.25 for each protein in a COG in < 90% of genomes			1	13.74735
OrthoMCL [24]†	Conservation	Orthologous clusters	Presence in an orthologous cluster	+ 1 for each protein in a COG in > = 90% of genomes in atleast one method	orthomcl_cluster1407|63	Present in > 90% of the genomes		
				−0.25 for each protein in a COG in < 90% of genomes				
LS-BSR [25]†	Conservation	Orthologous clusters	Presence in an orthologous cluster	+ 1 for each protein in a COG in > = 90% of genomes in atleast one method	63	Present in > 90% of the genomes		
				−0.25 for each protein in a COG in < 90% of genomes				
Attributor^c^	Function	Annotation & GO Terms	Annotation & GO Terms	+ 1 for each GO term in our surface exposed GO db	hypothetical_protein_domain_protein	No conclusive GO terms predicted	0	13.74735
				−1 for each GO term in our non-surface exposed GO db				
				+ 1 if presence of surface exposure keywords if predicted periplasmic				
^a^HMM: Hidden Markov Model. This component includes a collection of HMMs selected from the Pfam database for motifs associated with surface proteins.								
^b^IEDB: Immune Epitope Database and Analysis Resource								
^c^In house Perl/Python script								
^d^SSRs: simple sequence repeats								
*If any three of PSORTB, LipoP, SignalP and IEDB Database matches are all positive, weight is incremented by 2.						True	2	15.74735
^If all surface exposure tools fail a conclusive prediction, weight is decremented by 2						False	0	15.74735
†Each protein is given an additional 0.1 for > 90% presence in each of the clustering algorithms, Jaccard Clusters, PanOCT, OrthoMCL and LS-BSR, and penalized 0.5 for < 90% presence or absence of a cluster for each tool.						True	0.4	ReVac Score = 16.14735

Certain predicted features among outputs for these tools were not assigned weights as it was observed that their predictions may not accurately predict PVCs and hence, we were unable to assign a justified positive or negative weight. As such, PSORTB [13] suggests that the heparin binding protein (NHBA) from the Gram-negative bacterium *Neisseria meningitidis*, currently used in a multicomponent vaccine against meningococcal serogroup B, is localized exclusively in the periplasm. However, this is not consistent with experimental evidence that indicates the protein is exposed on the bacterial surface [23]. Thus, in the case of PSORTB predicted periplasmic proteins, no negative weight was assigned as some periplasmic predictions may be inaccurate or inconclusive such as in the case of NHBA. To account for this, we used multiple different tools for more accurate prediction of subcellular localization. Another example would be the case of pneumolysin from *Streptococcus pneumoniae*, an extracellular virulence factor [24]. PSORTB provided a strong extracellular prediction, however LipoP [25] suggested a cytoplasmic protein. Again, for the same reason, intracellular predictions of LipoP were not penalized. Wherever similar and other trends were noticed among other tools the weights were assigned and distributed using similar justifications (Described further in Methods). The remaining non-antigens had feature predictions and annotations consistent with intracellular localization across all tools. These were assigned negative weights for each tool suggesting an intracellular localization, which should be avoided as potential PVCs. A complete list of weights assigned, and the scoring scheme is presented in Table 2 and described in the Methods.

Tools comprising the antigenicity prediction features were all assigned positive weights relative to the proportion of antigenic regions within a protein and boosted if the presence of curated epitopes within the sequence was observed. Most of these tools operate by splitting an input protein sequence into individual peptides and analyzing them individually as potential epitopes; all proteins tend to have at least some antigenic regions. As a result, weights relative to percent of antigenic regions were assigned. Lastly, adverse features are those that should be avoided when choosing any PVC, such as repeat regions or similarity to host or commensal organism proteins. ReVac identified repeats within the *B. pertussis* pertactin transporter and the *N. meningitidis* heparin binding proteins. Such repeats suggest that these antigens may undergo slipped strand mispairing resulting in phase variation of the proteins, a negative feature of vaccine antigens [6]. Antigens with sequence repeats in either promoter or protein coding regions are therefore negatively penalized. Additionally, negative scores are given to antigens with features of similarity to host and commensal proteins, to avoid the negative effects of cross reactivity of an immunizing vaccine antigen. When both features were absent, ReVac attributes positive weights to the score to increase the ranks of the PVCs away from ones having these features.

As not all the tools implemented in ReVac could be run for our control dataset, such as those related to protein conservation across their many respective species and genomes, a lower score cutoff of 8 was chosen for these datasets. Using this threshold, 74 of the 136 Gram-positive antigens had a score of at least 8 with no non-antigens in the subset. 182 of 428 Gram-negative antigens had a score of at least 8 with 2 non-antigens in the subset (Table 1 and Additional file 4). It should be noted that given the breadth of species and the large number of validated antigens and non-antigens included in our control datasets, the scoring scheme we developed should be readily applicable to many bacterial pathogens. The scoring scheme can be applied iteratively to any number of new genomes being added to databases. We anticipate that the number of new genomes of interest will grow much faster than the experimental validation of new candidates that should be added to the control dataset. It is conceivable that many of the new candidates will harbor features similar to those already curated in our dataset and therefore will not change the scoring mechanism. However, when sufficient amounts of truly novel candidates become available in the future, an update to the scoring scheme could be released after some additional manual intervention. The simplest, systematic way of identifying the need for a new release will be to determine when a critical number of important validated candidates fail to be ranked near the top of the ReVac output.

### Application of ReVac to non-typeable *Haemophilus influenzae* (NTHi)

ReVac was run on 270 NTHi genomes, derived from sputum isolates obtained from a 20-year prospective study of adults with chronic obstructive pulmonary disease (COPD) [11, 26]. This dataset comprised 477,769 predicted protein encoding genes. Of these, 4477 proteins had scores that were not penalized more than 10% of their total score and grouped into ortholog clusters based on a consensus from 4 orthology prediction methods (See Methods). Each ortholog’s average score was calculated, as well as its range within its ortholog cluster. Clusters were then filtered based on the presence of at least 80% of proteins present in the lowly penalized 4477 dataset. This yielded 29 ortholog clusters which were high scoring, i.e. greater than 10, that included both core and dispensable genes of NTHi. Surveying this list, provides a highly prioritized selection of orthologs for consideration, and downstream experimental verification of vaccine candidacy potential. Candidates were prioritized based on a candidate cluster being highly conserved within the species (usually > 90%), and at least 80% of the proteins in the cluster being high scoring (to allow for some allelic variation), with a narrow score distribution. Based on these results, some of our top candidates include a Hemopexin transporter protein, involved in extracellular transport of hemopexin, and the outer membrane lipoprotein P4 (Table 3). The NTHi dataset of the analysis is provided in Additional file 2.

**Table 3:** Top candidates selected from ReVac’s output for NTHi and *M. catarrhalis* (M.W/pI represent molecular weights and isoelectric points)

Example locus tag	Amino acid length	M.W./pI	Annotation	Gene
NTHi				
84P48H1_01193	562	62.24/9.43	Hemopexin transporter	hxuB
84P8H1_00650	274	30.48/8.97	Lipoprotein E precursor	Hel (P4)
*M. catarrhalis*				
ADC73_RS07905	405	44.01/9.47	Hypothetical protein (porin family)	None
E9Y_00353	537	60.40/8.95	Protein of unknown function (DUF560)	None
M137P16B1_1805	344	38.37/8.95	Gram-negative porin protein	None
AO373_1452^a^	331	36.16/9.9	Ferric iron ABC transporter iron-binding protein	None

All the above candidates were surface exposed, predicted antigenic, conserved core proteins with low autoimmunity and no repeat regions. Further information about these candidates are available in Additional files 2 and 3 respectively. ^a^Present in *M. canis*

### Application of ReVac to *Moraxella catarrhalis*

The *Moraxella catarrhalis* dataset consisted of 69 genomes, 49 were obtained from NCBI and 20 were newly sequenced by our group. The latter were obtained from sputum isolates, from patients with COPD [11, 26, 27]. This dataset comprised 130,179 predicted protein encoding genes. Of these, 3995 met the maximum 10% penalization filter, and again filtered based on 80% presence in a cluster. Analyses resulted in 64 high scoring ortholog clusters (greater than 10) of core and dispensable genes of *M. catarrhalis*. Based on these results, top candidates identified were an iron transporter protein, a Gram-negative porin protein and 2 conserved-hypothetical proteins previously unstudied (Table 3). The *M. catarrhalis* dataset of the analysis is provided in Additional file 3.

### ReVac benchmarking and runtime

**Fig. 3:**
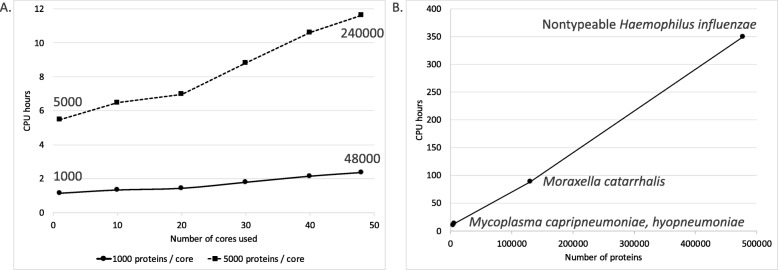
**a** An estimation of ReVac’s CPU time focused on its rate-limiting steps using batches of 1000 and 5000 proteins. Multiple runs (one for each time point on the figure) were submitted in succession on a single host, using increasing amounts of dedicated cores, each running the same batch of the respective 1000 (solid line) or 5000 proteins (dashed line). The total numbers of proteins analyzed using 1 and 48 cores are provided as labels for comparison to (**b**). **b** Real-life CPU time estimates derived from the entire ReVac workflow running on 150–300 compute clusters through Ergatis, each utilizing a single host in most cases

ReVac was run on 4 major datasets, namely, 2 control datasets of Gram-positive and Gram-negative antigens, to optimize the scoring algorithm for specific components, and our test datasets of *M. catarrhalis* and NTHi, to prioritize potential vaccine candidates. Two smaller datasets of mycoplasma proteins were also run as test data. For benchmarking purposes, proteins were batched into groups of 1000 and 5000 sequences and were run through ReVac’s most time-consuming commands to generate an estimate of CPU (Central Processing Unit) hours (Fig. 3a). These were run on an isolated, dedicated server with 2 CPUs (CPU model: Intel(R) Xeon(R) CPU E5–2690 v3 @ 2.60GHz, 256GB RAM), each with 12 cores with hyperthreading (approximately 48 virtual cores). An overall estimate was also made of ReVac’s runtime in hours for our test datasets, with actual runtime for the entire duration of a run, on multiple servers on a compute grid without CPU usage restrictions and competition with other, unrelated compute jobs potentially submitted to the same servers by other users (Fig. 3b) [10, 11, 28].

## Discussion

ReVac was developed to identify bacterial PVCs by evaluating multiple features based on ideal PVCs characteristics and homology to known PVCs. A major aim of ReVac’s development was to add multilayered-redundant analyses for most of the protein feature predictions used in parallel, in order to provide additional independent confidence in the respective feature predictions. The various tools employed encompass categories of essential features of PVCs; subcellular localization, antigenicity and immunogenicity, conservation and function, and genomic islands. ReVac builds on previously published reverse vaccinology pipelines and includes several major improvements: 1) the analyses of multiple genomes for a given species enabling assessment of conservation of PVCs (core genome) or, for instance, unique dispensable genome PVCs of hyper-virulent strains. 2) the evaluation of SSRs in both coding and upstream regions to assess phase variable expression of PVCs based on the presence of this nucleotide sequence feature (enabled by the use of multiple genomes). 3) the parallel analysis and summary total of the features for each protein of the input genomes, with positive scores for desirable features versus negative scores for undesirable features, reducing the false negative elimination of candidates. And 4) flexibility to prioritize or de-prioritize any feature based on organism-specific considerations of a vaccine development project, for example. The parallel scoring system allows resolution of a PVC ranked highly or poorly due to multiple features rather than just a few.

Reverse vaccinology pipelines, such as ReVac, are beneficial for the prioritization of PVCs for vaccine development against bacteria prior to experimentation. Providing short lists of ideal candidates to assay in vitro and in animal models is desirable and is especially powerful where animal models may not be well established. The human restricted pathogens *Moraxella catarrhalis* and non-typeable *Haemophilus influenzae* are examples of such bacteria with underdeveloped animal models [29]. The added innovative ability of ReVac to assess conservation and phase variability from multiple genomes is critical for these genetically diverse human pathogens [10, 11].

**Fig. 4:**
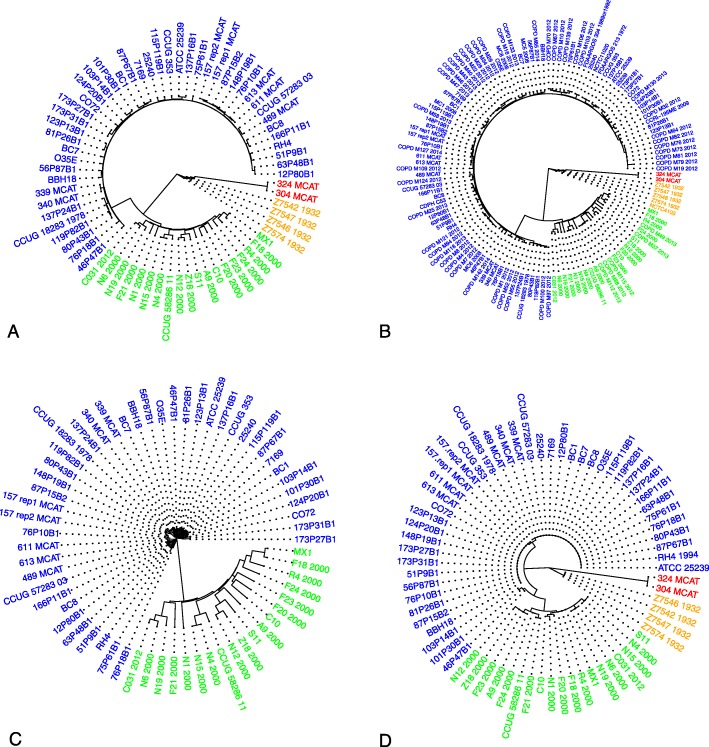
**a** Whole genome tree of the 69 *M. catarrhalis* genomes used in ReVac. The four clades seen are labeled as, blue-indicating a sero-resistant clade, green-indicating a sero-sensitive clade, orange-indicating older isolates of *M catarrhalis* dating to 1932, and red-indicating misannotated *M. canis* genomes from NCBI. **b** Whole genome tree of 128 currently available *M. catarrhalis* genomes on NCBI, maintains the same topology as 4A. **c** A protein alignment tree of one of ReVac’s top candidates, which separates sero-sensitive and sero-resistant clades, but is absent in the other two clades (also present in the respective clades of (**b**)). **d** A protein alignment tree of the candidate iron transporter that replicates the whole genome tree topology

**Fig. 5:**
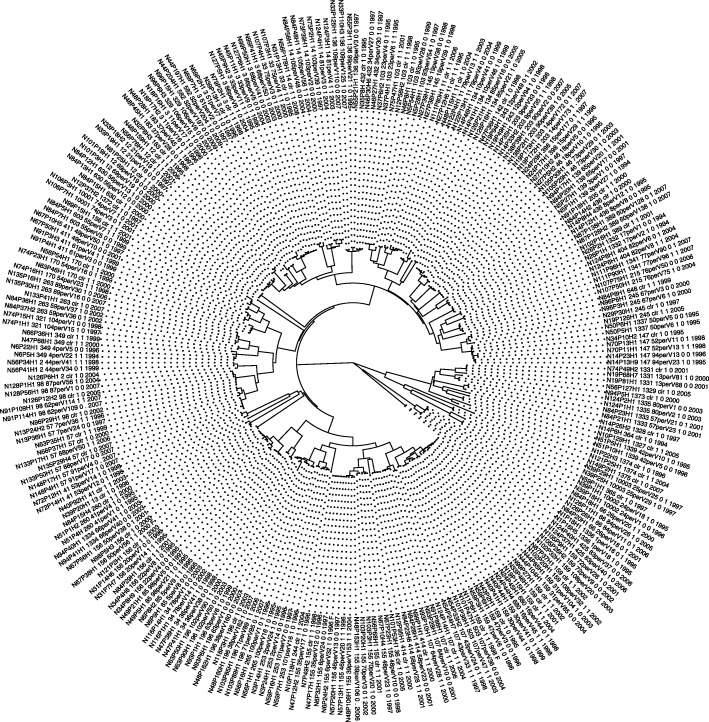
Whole genome tree of the 270 NTHi genomes used in ReVac

To provide an estimate of genetic variation among the *M. catarrhalis* genomes, we used MASH [30] to generate a pairwise genome distance matrix and associated phylogenetic trees (Figs. 4 and 5). We observed the formation of 4 distinct clades. Two of these clades were known to be separated based on sero-susceptibility of those strains (blue and green). Upon surveying the metadata of the genomes, we discovered that one of the other two clades clustered based on their date of isolation (orange) and the most distant was another species, *M. canis*, which was misannotated as *M. catarrhalis* in NCBI (National Center for Biotechnology Information). We attempted to identify similar relationships between strains in our NTHi genomes, however, there appears to be no correlation between the strains apart from clustering based on multilocus sequence types (MLST) as previously reported [11]. There was no clear genetic clustering based on clinical source of the strain, geography, duration of persistence, exacerbation vs. colonization, or year of isolation, of these strains. Upon investigation of some of our *M. catarrhalis* candidates we observed that some candidates, at the protein level, could recapitulate the same separation among clades as the whole genome tree (Fig. 4c and d). It is possible that these proteins are core drivers of inter-strain variation as not all protein clusters could replicate the whole genome tree.

Based on overall vaccine candidates from both NTHi and *M. catarrhalis* datasets, certain types of high scoring proteins are more prioritized over others. Hemolysins and TonB receptor proteins are identified as quality candidates in both species, however they are not included in our top candidates as they were not core proteins. Other types include various transporter proteins, outer membrane proteins and porins. This result is in part due to the outer membrane-surface exposed nature of these classes of proteins. However, ReVac’s comprehensive assessment of immunogenic epitope identification and cross-referencing of proteins against commensal organism’s genomes supports that there are dissimilar sequences of these proteins in the pathogen and its related commensal. Whether these proteins might be strong candidates in other species, will likely depend on their conservation across those species. The presence of redundant proteins to these will diminish their impact as vaccine candidates outside the two species considered here. Presence of similar types of proteins identified as vaccine candidates is interesting as both these species occupy similar niches in the host. In total, ReVac identified a set of surface exposed proteins in two exclusively human respiratory tract pathogens. The features of these proteins as pathogen-specific PVCs, provides strong rationale to experimentally validate the efficacy of the novel vaccine antigens against the pathogens.

## Conclusion

The identification of core vaccine candidates provides a path for vaccine development against prokaryotic pathogens. Use of essential-core genome components in vaccines reduces the impact of any selective pressure, which may be imposed on a pathogen through vaccine use. This phenomenon has been observed in the case of Group A *Streptococcus*, where several potentially effective vaccine candidates are not a part of the core genome [31]. Selection of these non-core candidates could result in non-vaccine strains dominating a now vacant niche. Another strength of ReVac’s prioritization of candidates on a cluster level, is that it allows for identification of clade-specific vaccine candidates. This approach could be powerful in situations where a species may be a commensal organism, but certain variants are pathogenic, such as in the case of *E. coli* [32]. A reverse vaccinology analysis at a whole pan-genome level will be able to identify and distinguish these candidates from core candidates.

All vaccine candidates are antigens, but not all antigens are effective vaccine candidates for various reasons. ReVac therefore prioritizes antigenic PVCs using predictions from multiple antigenicity and immunogenicity tools which are becoming more prominent as effective tools for identification of potentially novel epitope regions [33]. For example, certain antigens may induce more effective adaptive immune responses than others. It should be noted that ReVac is not an antigen predictor, but a workflow that ranks proteins by their vaccine candidacy potential. Follow-up in vitro and in vivo characterization will therefore be required to assess the validity of these PVCs as antigens. ReVac’s primary mandate is to help identify, as well as reduce the number of candidates that will have to be tested by providing the user with a ranked list of PVCs.

Here we have presented ReVac, a reverse vaccinology pipeline for the identification of bacterial protein PVCs from the input of one or more annotated bacterial genomes. ReVac’s implementation of a parallel scoring scheme of all proteins in the organisms’ proteome and summary scores minimizes elimination of false negative antigens due to potential errors in the prediction tools implemented. False positive identification of antigens is reduced by assigning lower scores to proteins associated with non-antigens, and with similarity to human host proteins as well as commensal organisms’ proteins. These multiple genome assessments performed in ReVac evaluate the conservation of PVCs and those subject to phase variable expression in a given population of bacterial strains to further reduce false positives. The compilation of these features integrated into ReVac’s pipeline was tested on sets of positive and negative control antigens to optimize the scoring algorithm. We used ReVac to identify PVCs for the human-restricted pathogens *M. catarrhalis* and NTHi. We identified both known and novel PVCs for each bacterium, supporting the efficacy of ReVac to identify experimentally proven PVCs. Furthermore, the identification of previously uncharacterized proteins as PVCs shows the benefit of ReVac to identify novel protein targets to investigate in future studies. ReVac can be used to identify PVCs of current bacterial pathogens where vaccines are not currently developed, and can also be used to quickly identify PVCs of emerging pathogens as sequences become available.

## Methods

ReVac and all its tools were built and integrated together using the open-source Ergatis workflow management system (available at http://ergatis.sourceforge.net) [14]. Ergatis allows the user to monitor bioinformatic pipelines, such as ReVac, through its user interface, and allows the parallelization of several analyses on distributed computer clusters [14]. Individual tools can be installed as Ergatis components and their analyses then parallelized, for any bioinformatic pipeline. For these reasons, Ergatis was chosen as a streamlined way of performing all ReVac’s computationally intensive multi-genome analyses while allowing for easy access to output data and monitoring.

Here, we provide a description of all the tools incorporated in the ReVac pipeline, including those involved in file format conversions. The complete scoring weight scheme for each component is described in Methods. The rationale for assigning different weights to specific components was derived from the results of ReVac runs on our control datasets of known antigens and non-antigens acquired from various antigen databases from multiple bacterial species (Additional file 1 and Fig. 1). Components are grouped into broad categories (Table 2), as follows:

1. Subcellular Localization
  - PSORTb 3.0 [13] – A bacterial protein subcellular localization (SCL) predictor geared for all prokaryotes, including archaea and bacteria with typical and atypical membrane/cell wall topologies [13]. PSORTb 3.0 assigns a protein one of seven possible SCLs, namely, Extracellular, Cell Wall, Outer Membrane, Periplasmic, Cytoplasmic Membrane, Cytoplasmic & Unknown. We ran PSORTb 3.0 on control datasets, 69 *M. catarrhalis* and 270 NTHi genomes using default parameters with an overall cut off set at a value of 7.5 for the overall prediction score. Proteins that were predicted to localize at multiple sites were also included if their cumulative score was above 7.5. We used the long output-type option provided by PSORTb and predictions are provided in the summary table. Each protein was given a +  1 towards its overall score if it wholly or partially localized to the outer membrane, cell wall or was predicted to be extracellular. If the protein was predicted to localize only to the cytoplasm or cytoplasmic membrane it was given a − 1. A prediction of Periplasmic was not scored as proteins from control datasets showed that some outer membrane proteins were often miscalled as periplasmic.
  - LipoP [25] – A hidden Markov model (HMM)-based tool to predict lipoprotein signal peptides in Gram-negative Eubacteria, able to distinguish between lipoproteins Signal peptidase II (SPase)-cleaved proteins, SPaseI-cleaved proteins, cytoplasmic proteins, and transmembrane proteins [25]. Although it was developed for Gram-negatives, it has shown effective performance on the prediction of Gram-positive bacterial lipoproteins [25]. We ran LipoP using default parameters with a cutoff of − 3. Each protein was given a +  1 towards its overall score if it was predicted to be a signal peptidase or had transmembrane helices.
  - TMHMM 2.0 [34] – A membrane protein topology prediction method, based on an HMM [34]. It predicts the number of transmembrane helices present in a protein sequence. TMHMM was run using default parameters and predicted number of helices are displayed in the summary table. Each protein was given + 0.5 if it had a single helix. It is not scored for 2 helices and penalized for more than 2 helices (− 0.2 for 3 and − 2 for 4 or more), as such proteins are harder to purify and less likely to be accessible on the cell surface. If the protein was predicted partially cytoplasmic or in the cytoplasmic membrane, it was penalized an additional − 2.
  - SignalP 4.1 [35] – A program developed for the prediction of signal peptides from amino acid sequences that are targeted to the secretory pathway [35]. We ran SignalP using the ‘best’ option for prediction allowing a truncated length of 70 for the peptides. We also disabled its graphical output as we were only interested in presence/absence of signal peptides. Its raw output provides coordinates of a protein’s signal peptide, which we then used to map back the actual signal peptide sequence for the summary table. Each protein was given a +  1 if it had a signal peptide.
  - SPAAN [36] – An artificial neural network developed to predict the probability of a protein being an adhesin. Adhesins are surface exposed proteins which mediate the adhesion of microbial pathogens to host cells [36]. We ran SPAAN using a cutoff of 7. Each protein was given a +  1 if it was a predicted adhesin.
  - HMMPFAM 3.0 [37] – A HMMER 3.0, HMM based tool, which queries a given amino acid sequence against multiple relevant subsets of TIGRFAM and PFAM databases [38, 39] namely, surface exposed, signal proteins, secreted proteins, and bacteriocins. This database of motifs was developed from the TIGRfam v15.0 and Pfam v31.0 databases, manually filtered based on keywords in their descriptions, which were relevant to surface exposure. We used default noise threshold cutoffs for all our HMM runs. Each protein was given a +  0.5 for any positive hit against motifs in this database.
2. Antigenicity & Immunogenicity
  - Antigenic (http://www.bioinformatics.nl/cgi-bin/emboss/antigenic) – An EMBOSS package utilizing the method of Kolaskar and Tongaonkar to predict antigenic determinants in proteins [40]. This tool outputs the antigenic peptide regions within a protein. We used a minimum peptide length of 9. Overlapping peptides are merged into larger regions to calculate overall percent coverage of the whole protein. The total number of antigenic peptides and their percent coverage of the protein are presented in the summary table. Each protein is given + 0.5 for having antigenic regions and an additional score of the ratio of its antigenic regions over its amino acid length.
  - MHC Class I [16] – An MHC I binding prediction tool acquired from the Immune Epitope Database & Analysis Resource (IEDB), containing 8 different peptide binding prediction methods. Of the 8, we have applied the consensus method for our analysis, using default parameters, applied across all 78 human MHC I alleles available for this method. The raw outputs assign a rank for each peptide of length 9, from a protein, with a sliding window of a single amino acid. The 99th percentile peptide sequences across all 78 alleles are selected, sorted based on coordinates within a protein, and then tiled together to produce a consensus MHC I binding region. The total number of binding peptides, the number of alleles they bind, their total consensus region, and their percent coverage, are presented in the summary table. Each protein is given a score of the ratio of its binding regions over its amino acid length, if its binding regions cover 80–95% of the protein. If that ratio is greater than 95% it is given a +  1. The weight is also boosted by the ratio of binding peptides of length 9, over the total number of peptides possible for a given protein with a sliding window of 1. The rationale for this being that when a protein is processed for MHC loading, the more peptides that can bind to, from all that could be generated, the better the overall binding of the protein.
  - MHC Class II [16] – Also acquired from IEDB, this tool comprises of 5 methods for MHC peptide binding prediction. Here again, we have chosen the consensus method, using default parameters, across all 63 human MHC II alleles available. Like the MHC Class I component, the raw outputs assign a rank for each peptide of length 15, from a protein, with a sliding window of a single amino acid. Here, the 95th percentile is selected as MHC class II binding is less efficient due to the MHC II molecules being open at the ends of peptide the binding region. The post processing of the raw outputs is handled the same as in MHC Class I. Each protein is scored like the scheme followed in MHC Class I.
  - NetCTLpan [16] – Another MHC I binding predictor acquired from IEDB included in our analysis. This tool makes its predictions by also considering the precursory steps involved in MHC peptide binding, such as proteasome cleavage and TAP (Transporter associated with antigen processing). Analysis was conducted across 12 MHC allele super-types available in this package, using a peptide length of 9 with default parameters for thresholds, TAP & cleavage weightage. The post processing of the raw outputs is handled the same as in MHC Class I. Each protein is scored like the scheme followed in MHC Class I.
  - B Cell Pred [16] – A linear B cell epitope predictor also procured from IEDB. It scores amino acid residues using 6 different scale-based methods. We have applied all 6 methods in our analysis and only those peptide sequences which are predicted to be epitopes across all methods are considered. Analysis was conducted using default cutoffs and parameters across all 6 methods using a peptide length of 7. As in the case of MHC I, peptide epitopes from all 6 methods are tiled together to form consensus predicted B-cell epitope regions. Each protein is given a score of the ratio of its binding regions over its amino acid length. Here again it is boosted by the ratio of binding peptides of length 7, over the total number of peptides possible for a given protein with a sliding window of 1.
  - Immunogenicity [16] – A Class I Immunogenicity tool from IEDB, that uses amino acid properties as well as their position within the peptide to predict the immunogenicity of a peptide-MHC (pMHC) complex. The peptides predicted to be effective MHC I binders from MHC Class I & NetCTLpan (the 99th percentile) are passed as inputs here and run using default parameters. Each protein is scored similar to the scheme followed in MHC Class I, but scores for 20% coverage or more, as not all MHC bound peptides induce an immune response.
  - BLAT [15, 16] – Using the BLAST-like Local Alignment Tool, amino acid sequences are compared to a database of experimentally curated epitope sequences acquired from IEDB. Using the BLAST output type and default parameters, protein regions mapping to curated epitopes are again tiled to get consensus epitope regions and their percent coverage. Each protein is given a score of the ratio of its homologous regions over its amino acid length. If that ratio is greater than 70% it is given another + 1. Here if the protein is positive for surface exposure in any 3 among PSORTb, LipoP, SignalP and IEDB, it is given a + 2 as this pattern was observed frequently in positive control datasets.
3. Conservation & Function
  - Jaccard Clusters of Orthologous Genes (COG) Analysis [41] – A two-phase protein clustering algorithm, used to generate protein paralog and ortholog clusters. It parses an all-v-all BLAST [42] output of whole genomes, after which a Jaccard similarity coefficient is calculated for every pair of proteins. Then it performs a bidirectional best hit analysis on the paralog clusters generated by the first phase of the algorithm, rather than on individual proteins to call its orthologs [41]. Each protein belonging to a COG that is present in 90% of the genomes provided receives + 1 (in at least one of the conservation methods).
  - PanOCT [43] – A tool for pan-genomic analysis of closely related prokaryotic species or strains using conserved gene neighborhood information to separate recently diverged paralogs into orthologous clusters [43]. We elected to use 90% conservation as our cut-off and ran the analysis at default parameters. The PanOCT component was adapted to use a list of GFF files, a multi-FASTA file of all amino acid sequences and an all-v-all BLAST file run using the -m8 output option, to generate its config file and then perform clustering. Each protein belonging to a COG that is present in 90% of the genomes provided receives + 1 (in at least one of the conservation methods).
  - OrthoMCL [19] – This program implements a scalable method for constructing orthologous groups using a Markov Cluster algorithm to group (putative) orthologs and paralogs [19]. Analysis makes use of a MySQL database to which data is stored during the clustering process, using an all-v-all m8 BLAST file and a multi-FASTA of all proteins, run at default parameters. Each protein belonging to a COG that is present in 90% of the genomes provided receives + 1 (in at least one of the conservation methods).
  - LS-BSR [44] – Large Scale Blast Score Ratio (LS-BSR) compares the genetic content of hundreds to thousands of bacterial genomes and returns a matrix that describes the relatedness of all coding sequences (CDSs) in all genomes surveyed [44]. LS-BSR was run at default parameters, using a list file of strain specific DNA sequences of predicted genes to produce a BSR matrix of proteins that cluster with each other. Default cutoff is 0.7 but we elected to use 0.6 as some proteins which clustered together in other tools as core, were barely missing the default cutoff. Each protein belonging to a COG that is present in 90% of the genomes provided, receives + 1 (in at least one of the conservation methods). Here, if a given protein is shown to cluster in 90% of the genomes across multiple methods (described above), it is given an additional + 0.1 for each of those clustering methods.
  - Attributor – An in-house developed python script which refreshes annotations for a FASTA file of amino acid sequences, and assigns GO terms wherever applicable. It accepts inputs from TMHMM, LipoP, RAPSearch2 and HMMPFAM to call a specific annotation for a given protein. Each protein that has a GO term belonging to our database of surface exposed GO terms, is scored + 1 for each GO term, and − 1 for each GO term falling in our non-surface exposed GO term database, if none were identified in our surface exposed GO terms. Both GO term databases were constructed from the prokaryotic subset of GO terms, and then manually filtered for relevant surface exposed GO annotations, as well as Attributor GO term predictions for experimentally curated surface and non-surface exposed proteins acquired from the ePSORTB database. Here we also looked to rescue PSORTb predictions calling periplasmic, when proteins are surface exposed based on attributor, and scoring an additional + 2.
4. Exclusion Features
  - Autoimmunity [22] – Autoimmunity is a Perl script taken form NERVE [22] and adapted to run on Ergatis. It uses BLAST to compare each amino acid sequence as a query against the human proteome, allowing for 3 substitutions and 1 mismatch, over a minimum peptide length of 9. Raw outputs are again tiled together to give consensus regions of autoimmunity against Human proteins. Each protein that doesn’t map to the human proteome is given a +  1. Any protein that has a positive hit is penalized by 2 times the ratio of its homologous regions over its amino acid length. If that ratio is greater than 20% it is penalized another − 2.
  - Autoimmunity Commensals [22] – This component is an adaptation of the one above that runs against a non-redundant database of a commensal organism of choice. In our case, since we were looking at NTHi & *M. catarrhalis*, the most closely related species selected were *H. haemolyticus* and *M. bovis*, respectively. The database of non-redundant protein amino acid sequences was made from 13 strains of *H. haemolyticus* acquired from NCBI and then clustered using OrthoMCL, for NTHi. However, only one strain of *M. bovis* was available on NCBI. Each protein is scored the same as autoimmunity.
  - SSR_Finder [45] – SSR_finder is a script developed by Siena et al. [45] which looks for Simple Sequence Repeats, up to 10 base pairs in length, in DNA coding sequences and 500 bp upstream of the gene. SSRs have been shown to contribute to phase variation of proteins allowing generation of different protein isoforms and mediating on/off translational switching through frameshifts [45]. It scans through a list of multi contig DNA fasta files for such SSRs. Each gene is given a +  1 for absence of an SSR. Presence of a repeat is penalized − 0.5 for each. An additional − 0.25 is received if the repeat is in promoter region, − 0.5 if the repeat has the potential to cause a frame shift, as well as − 0.01 times the total length of the repeat.
  - SSR_Finder_Protein [45] – The above script was adapted to run on protein sequences looking for repeats, up to 20 amino acids in length, which would allow conformational changes in the protein. Each protein is penalized − 0.2 for each protein repeat, up to a maximum penalty of − 1.
5. Genomic Islands
  - IslandPath [46] – IslandPath is a tool developed for the detection of genes of potential horizontal transfer origin known as Genomic Islands, in prokaryotes [46]. Islandpath accepts NCBI Protein Table (ptt) files of the proteome as well as protein & coding sequence FASTA files with their coordinates to identify possible genomic islands. Each protein present within a genomic island is penalized − 0.5.
6. Foundation Components
  Input data that is passed through ReVac, requires a multitude of formats as per the component being invoked, as well as supplementary data from other predictive components. ReVac uses the following foundation components, which are provided as a part of the Ergatis workflow [14].
  - Genbank2bsml – Accepts standard GenBank file formats (.gbk) for conversion to Bioinformatic Sequence Markup Language (BSML), which is an Ergatis standard file format for data in addition to the more common FASTA files.
  - Bsml2fasta – Accepts the BSML inputs from Genbank2bsml for conversion to FASTA files. This component allows for the generation of single sequence FASTA files or a larger multi-sequence file, providing the option of generating numerical sequence IDs for each sequence as well filtering files to contain either solely nucleotide or amino acid sequences.
  - Split_multifasta – Splits the multi-sequence file(s) generated by Bsml2fasta into smaller files containing a user-defined number of sequences, for distribution onto the grid for the later components.
  - Bsml2ptt – Allows conversion of the BSML files into a tab delimited ptt file. Currently only utilized in the IslandPath component.
  - Extract_CDS_Features – An in-house developed script to parse Genbank files and extract all features relevant to any coding sequences present. These include contig ID, coordinates, amino acid length, estimated molecular weight, isoelectric point, gene name, and annotation.
  - Formatdb/Xdformat – These components format and index whole proteome FASTA files for all-v-all BLAST searches.
  - NCBI-BLASTP/WU-BLASTP – Different components require different types of BLAST outputs; hence both are available for use in ReVac.
  - RAPSearch2 [47] – A new upgraded protein similarity search tool for next generation sequencing data, which scans the latest UniRef and UniProt databases for use in Attributor.

The current ReVac workflow package available on github (https://github.com/admelloGithub/ReVac-package) is designed to work through the Ergatis management system, after all required tools and dependencies are installed.

### Phylogenetic tree construction

We used MASH [30] using default settings to acquire pairwise distance matrices for our whole genome trees. These we then converted into Newick tree files using the Neighbor-Joining method in MEGA7 [48] and unrooted tree figures were constructed in R studio using the APE package [49]. For our protein ortholog trees, the amino acid FASTA sequences were acquired from the ReVac’s orthology components outputs and aligned using ClustalW in MEGA7 with default parameters, and Newick tree files generated using the Neighbor-Joining method, for use in R studio with the Ape package.

## Supplementary information

**Additional file 1.** Control datasets used for development of the scoring scheme.

**Additional file 2.** Nontypeable *Haemophilus influenzae* dataset.

**Additional file 3. ***Moraxella catarrhalis* dataset.

**Additional file 4.** Examples of control proteins used for development of the scoring scheme.

## Data Availability

The datasets supporting the conclusions of this article are included within the article and its supplementary tables and additional files.

## Abbreviations

BSML: Bioinformatic Sequence Markup Language
BSR: Blast Score Ratio
CDSs: Coding sequences
COG: Clusters of orthologous genes
COPD: Chronic obstructive pulmonary disease
CPU: Central Processing Unit
GFF: General Feature Format
GI: Genomic islands
GO: Gene Ontology
HMM: Hidden Markov Model
IDs: Protein identifiers
IEDB: Immune Epitope Database
M.W: Molecular weight
MHC: Major Histocompatibility Complex
MLST: Multilocus sequence type
NCBI: National Center for Biotechnology Information
NHBA: *Neisseria* Heparin Binding Antigen
NTHi: Nontypeable *Haemophilus influenzae*
pI: Isoelectric point
ptt: NCBI Protein Table
PVC: Potential vaccine candidates
RAM: Random Access Memory
SCL: Subcellular localization
SPase: Signal peptidase
SSRs: Simple sequence repeats
TAP: Transporter associated with antigen processing
